# Principles of chemical geometry underlying chiral selectivity in RNA minihelix aminoacylation

**DOI:** 10.1093/nar/gky909

**Published:** 2018-10-15

**Authors:** Tadashi Ando, Shunichi Takahashi, Koji Tamura

**Affiliations:** 1 Department of Applied Electronics, Tokyo University of Science, 6-3-1 Niijuku, Katsushika-ku, Tokyo 125-8585, Japan; 2 Research Institute for Science and Technology, Tokyo University of Science, 2641 Yamazaki, Noda, Chiba 278-8510, Japan; 3 Department of Biological Science and Technology, Tokyo University of Science, 6-3-1 Niijuku, Katsushika-ku, Tokyo 125-8585, Japan

## Abstract

The origin of homochirality in L-amino acid in proteins is one of the mysteries of the evolution of life. Experimental studies show that a non-enzymatic aminoacylation reaction of an RNA minihelix has a preference for L-amino acid over D-amino acid. The reaction initiates by approaching of a 3′-oxygen of the RNA minihelix to the carbonyl carbon of an aminoacyl phosphate oligonucleotide. Here, employing molecular dynamics simulations, we examined the possible mechanisms that determine this chiral selectivity. The simulation system adopted a geometry required for the chemical reaction to occur more frequently with L-alanine than that with D-alanine. For L-alanine, the structure with this geometry was formed by a combination of stable dihedral angles along alanyl phosphate backbone with a canonical RNA structure, where the methyl group of alanine was placed on the opposite side of the approaching 3′-hydroxyl group with respect to the carbonyl plane. For D-alanine, the methyl group and the 3′-hydroxyl group were placed on the same side with respect to the carbonyl plane, which significantly decreased its ability to approach 3′-oxygen close to the carbonyl carbon compared to L-alanine. The mechanism suggested herein can explain experimentally observed chiral preferences.

## INTRODUCTION

Except glycine, all α-amino acids exist as D- and L-optical isomers. However, proteins synthesized on the ribosome consist exclusively of L-amino acids. In current biological systems, an enzyme called aminoacyl-tRNA synthetase (aaRS) attaches a correct L-amino acid to a specific transfer RNA (tRNA) molecule to form an aminoacyl-tRNA, which ensures the utilization of L-amino acids in protein synthesis. The origin of this homochirality is one of the mysteries of the evolution of life (1). It is plausible that a primitive protein synthesis machinery first developed during the RNA world (2,3). Tamura and Schimmel showed that aminoacylation of an RNA minihelix has a chiral preference for L-amino acid over D-amino acid with neither protein nor ribozyme (4,5). The model formation was inspired by the fact that contemporary aaRSs use aminoacyl-adenylates as intermediates for aminoacylation of tRNAs (6–8). The aminoacyl phosphate oligonucleotide used in the model is a mimic of aminoacyl-adenylate, and Tamura and Schimmel found out how to bring the activated amino acid in close proximity to the amino acid attachment site of RNA minihelix without any proteins, considering that a primitive aminoacylation system would have appeared in the RNA world (4). The minihelix corresponds to the acceptor stem and TΨC stem/loop of tRNA and is thought to be a progenitor of modern tRNA (9).

**Figure 1.**
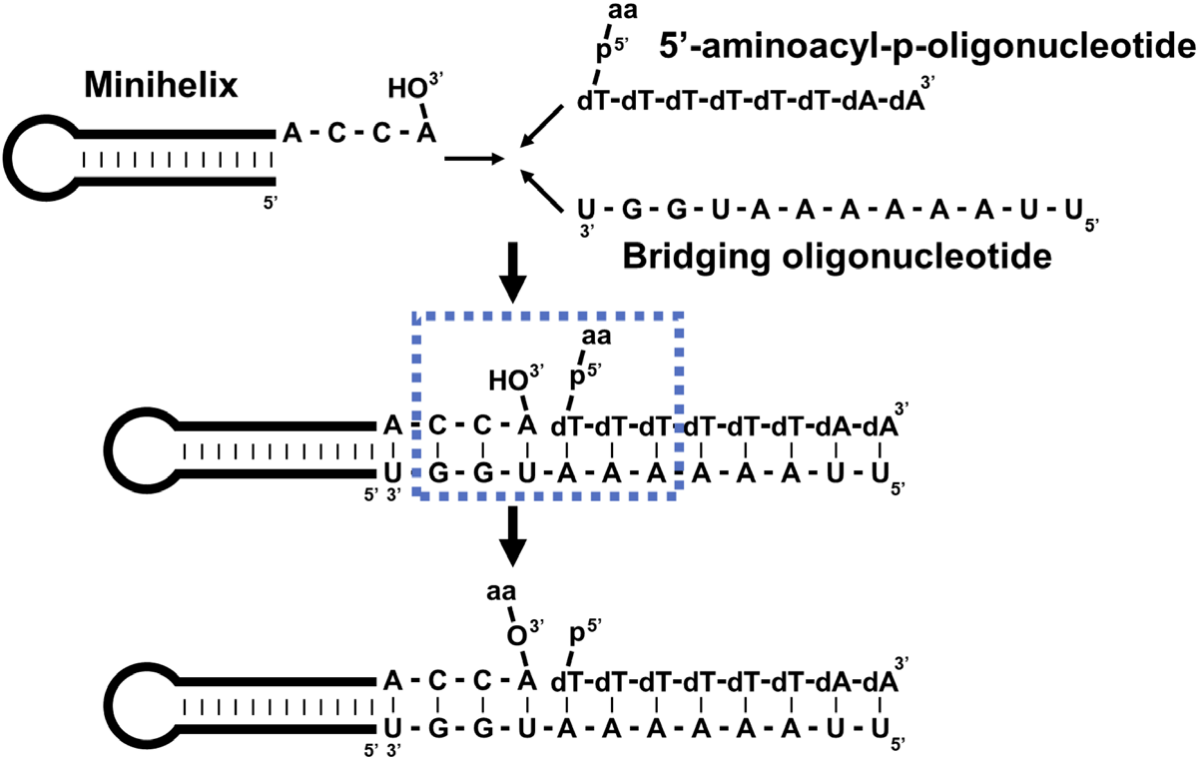
Schematic view of aminoacylation reaction with an RNA minihelix system. Minihelix, bridging oligonucleotide and 5′-aminoacyl-p-(dT)_6_(dA)_2_ were mixed together to transfer aminoacyl moiety from 5′-phosphate of the dT of oligonucleotide to 3′-hydroxyl group of the A, where dT and dA are deoxythymine and deoxyadenosine, respectively; ‘aa’ represents amino acid. The nucleotide sequences surrounded by the blue dotted rectangle with aa of alanine were modeled in this simulation study. Nucleotide sequences of the model were numbered as C_1_C_2_A_3_dT_4_dT_5_dT_6_ for the primary strand and A_7_A_8_A_9_U_10_G_11_G_12_ for the complementary strand.

In the simplified model, RNA aminoacylation is a thermodynamically favorable ‘downhill’ reaction: a high-energy aminoacyl phosphate donor nucleotide (mimic of aminoacyl-adenylate) at a 5′-terminus is used to form a lower energy aminoacyl carboxyl ester linkage at a 3′-terminus with the help of a bridging nucleotide (Figure 1) (10). The free energy of hydrolysis of an aminoacyl phosphate is ∼3 kcal/mol greater than that of an aminoacyl carboxyl ester (11). The reaction is site-specific at 3′-OH (4,5), and the nucleophile O from 3′-OH in a terminal RNA attacks C in the carbonyl group C=O in the acyl phosphate linkage. The chiral selectivity was ∼4-fold, at least in the cases of several amino acids with different degrees of bulkiness, e.g. Ala, Leu and Phe (4). Their findings suggest that L-amino acid homochirality of proteins could be determined by preexisting homochirality of the D-ribose RNA (4,5). Since aminoacylation of tRNA is the first step of protein synthesis and establishes the genetic code (12), chiral-selective aminoacylation of the RNA minihelix is a potential progenitor for modern tRNA-based protein synthesis using L-amino acids.

Tamura and Schimmel performed a series of experiments to propose a model that differences of steric clash of the L- and D-amino acid side chains give rise to the chiral selectivity (5,10). For validating their model, structural information of the RNA minihelix at the atomic scale is crucial: this provides us new insights to conduct further experiments using kinetic, biochemical and computational approaches for revealing the mechanism. However, because of the extremely unstable nature of aminoacyl phosphate nucleotide, solving atomic structures of the molecule remains challenging.

**Figure 2.**
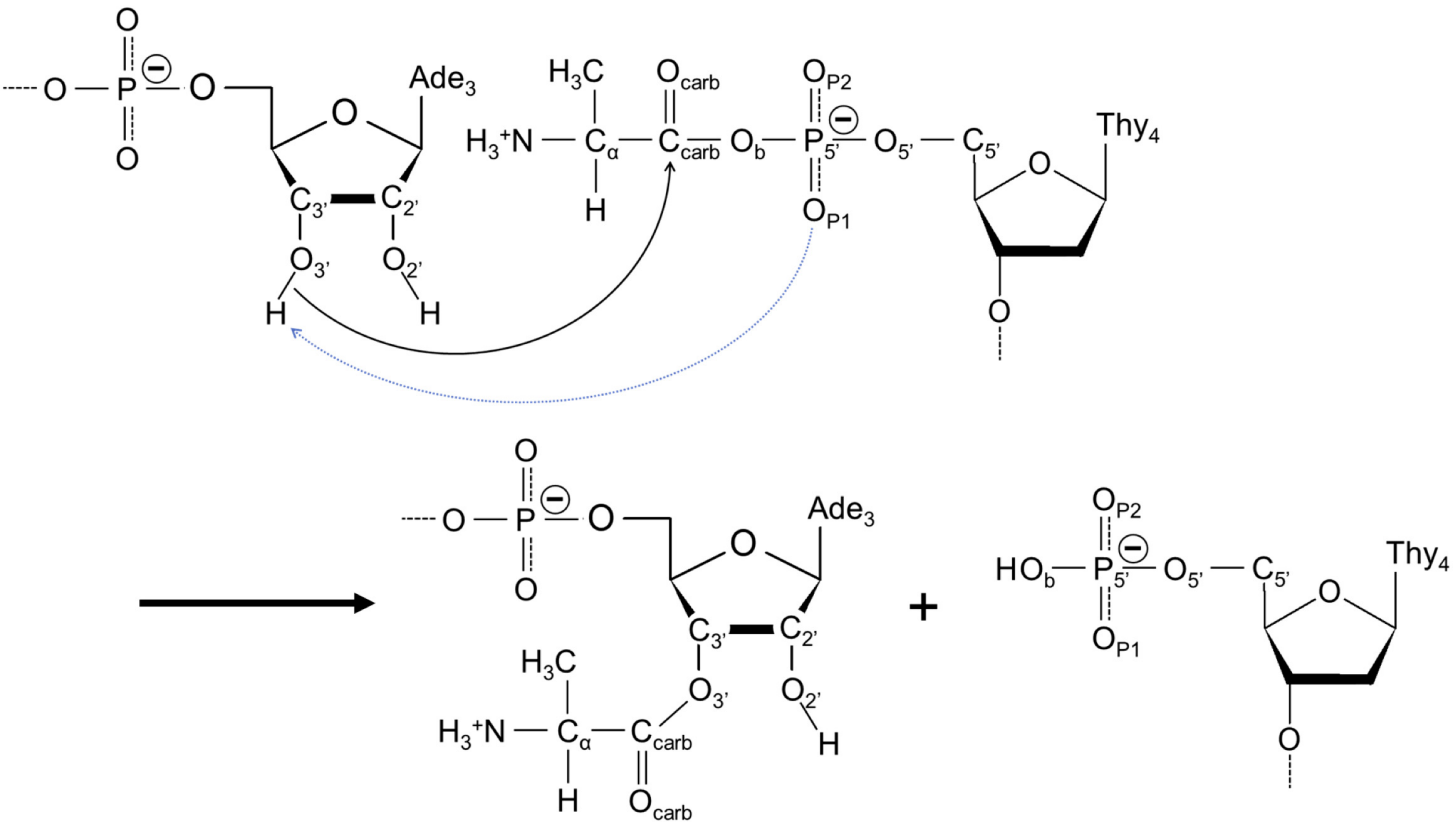
Reaction schemes for the aminoacylation found in the RNA minihelix. The blue dotted arrow represents a deprotonation of 3′-hydroxyl group proposed by SAC mechanism. Abbreviations of atoms used in this study are also described. Although O_P1_ atom acts as a base in this figure, O_P2_ atom also has a potential to work as a base.

Here, we employed molecular dynamics (MD) simulations to investigate chiral-selective aminoacylation mechanisms in the model RNA minihelix at the atomic scale. The system using a periodate oxidized aminoacylation-unfeasible minihelix with 5′-L- or D-aminoacyl phosphate nucleotide, and the bridging nucleotide showed no significant difference in the hydrolysis of amino acids (4). Therefore, we focused on the probability of properly approaching nucleophile 3′-O to the carbonyl carbon of the aminoacyl group (Figures 1 and 2), which can be estimated by classical MD simulations. Atomic structures with six base pairs centered at the reaction site were modeled, where L-Ala or D-Ala is covalently attached at 5′-phosphate via an acyl phosphate linkage (region indicated by blue dotted rectangle in Figure 1). The force field parameters, such as van der Waals radii, their energy strengths and atomic charges required for MD simulations were identical in the L-Ala and D-Ala systems. It was determined how often the systems with L-Ala and D-Ala adopt a conformation with a ‘reactive geometry’ during the trajectories obtained by MD simulations. This geometry was defined as optimal for the reaction at active sites based on knowledge of physical chemistry and an aminoacylation mechanism found in aaRSs. Based on the obtained structures, we propose the possible mechanism underlying the chiral-selective aminoacylation of the RNA minihelix.

## MATERIALS AND METHODS

### Initial structure modeling

Figure 1 shows the scheme of aminoacylation of RNA minihelix. We utilized the molecules inside the blue dotted rectangle for modeling. Initial double-stranded nucleotides structures were constructed using the UCSF Chimera (13) as follows. (i) A 3D structure of A-form RNA double helix was built with the nucleotide sequence shown, where dT was replaced by U. (ii) The last three U in the primary strand were changed to dT by manually editing the structure file. (iii) The ester bond between the 3′-oxygen atom in A_3_ and the phosphorus atom in the primary strand of dT_4_ (A_3_-(3′-O)) – (5′-P-dT_4_) was deleted to attach a hydrogen atom to 3′-O and L-Ala or D-Ala to 5′-P, respectively. These manipulations resulted in A_3_-(3′-OH) and L/D-Ala-(5′-P)-dT_4_ structures.

### Force field parameters for the RNA minihelix model

For DNA and RNA parts, Amber OL15 (14–16) and OL3 (17,18) force fields were used, respectively. The alanylated dT_4_ has NH_3_^+^-termini, where the chemical structure of the Ala is the same as the N-terminal Ala in usual proteins except for the ester oxygen atom linked to a phosphate atom. Therefore, the force field parameters for N-terminal Ala in Amber ff14SB force field were used for the alanylated dT_4_. For the ester oxygen atom, Amber atom-type was assigned to OS. The atomic charge was set to −0.3079, which gave a total charge of zero for Ala-dT. The parameters for bond, angle and dihedral involving the ester oxygen atom were found in Amber ff14SB. The TIP3P model was used for water.

### Simulation systems, conditions, and analysis

All simulations were performed using the AMBER 16 software package (19). Energy minimization was performed for the initial structures generated through the procedure described above in order to remove non-physical contacts/interactions between atoms in Ala and in DNA/RNA. For this purpose, atoms in DNA/RNA were restrained at their initial positions using a harmonic restraint with force a constant of 500 kcal/mol/Å^2^. The dielectric constant was set to 20, and the cut off length was set to 9 Å. The structures were minimized using 2000 iterations, of which the first 1000 iterations used the steepest descent (SD) method, and the remaining steps used the conjugate gradient (CG) method. To generate various initial conformations of the L-Ala and D-Ala systems, MD simulation at 600 K *in vacuo* were performed for 30 ps. The structure was sampled every 1 ps, thus a total of 30 structures for each system was passed to the next process for building fully solvated systems.

30 L-Ala and D-Ala modeled RNA minihelix structures obtained *in vacuo* simulations were solvated in cubic boxes containing ∼2900 water molecules and 10 Mg^2+^, 62 Cl^−^ and 52 Na^+^ ions. The net charge of the system was zero. The side length of the box was ∼50 Å. The NaCl concentration was roughly 1 M, which mimics the experimental condition (4,5). The solvated systems were minimized using the SD method for 1000 iterations, followed by applying the CG method for the next 1000 iterations with positional restraints applied to the DNA/RNA molecules. Minimization calculations of the entire system were performed with the SD and CG methods for the same number of iterations described above, where five distances between non-hydrogen atoms involving hydrogen bonds in base-pairs C_1_-G_12_ and dT_6_-A_7_ were harmonically restrained with a force constant of 100 kcal/mol/Å^2^ to prevent unfolding at both termini of the RNA/DNA fragment. These restraints were applied throughout the MD simulations described below. The minimized systems were heated from 1 to 300 K for 100 ps at constant volume conditions. Following this, to equilibrate the systems, *NVT* runs were performed for 100 ps, followed by *NPT* runs for 100 ps. The equilibrated systems were now subjected to a production run at *NPT* conditions for 20 ns with different random seeds. Therefore, a total of 30 production runs for 20 ns were performed in each system from different initial structures and different random seeds. One lakh and twenty thousand structures were sampled every 5 ps and were used for analysis of each system. Bonds involving hydrogen were constrained using the SHAKE algorithm (20) for all MD simulations, allowing a 2-fs time step. Temperature was controlled with the Langevin thermostat (21) with a 1.0 ps^−1^ collision frequency. Pressure was regulated with the Berendsen algorithm (22) at 1 atm with a pressure relaxation time of 1.0 ps. MD simulations with *NPT* conditions were performed using a GPU.

Analyses were conducted by CPPTRAJ analysis routines included in the AMBER package (23).

### Geometric requirements for the reaction

Figure 2 shows a general reaction scheme describing the transfer of an aminoacyl moiety from aminoacyl phosphate oligonucleotide to the RNA minihelix. A base is thought to deprotonate 3′-OH of the terminal adenosine of the RNA, generating an R-O^−^ nucleophile. This alkoxy anion then attacks the α-carboxylate carbon of aminoacyl phosphate oligonucleotide, resulting in transfer of the aminoacyl moiety to the 3′-O. The mechanism of aminoacyl transfer to tRNAs by aaRSs has been actively investigated by biochemical/structural (24,25) and theoretical/computational studies (26–28). Currently, for many of aaRSs, the transfer mechanism of the aminoacyl moiety can be explained well by substrate-assisted catalysis (SAC) (29); a functional group in a substrate contributes to enzymatic catalysis. In aaRSs, non-bridging phosphate oxygens of aminoacyl-adenylate are able to act as the base to deprotonate the hydroxyl groups.

Principles of physical organic chemistry and the SAC mechanism proposed for aaRSs give important clues regarding the geometry required for chiral-selective aminoacylation of the RNA minihelix. Here, we list the geometric requirements for the reaction.

**Figure 3.**
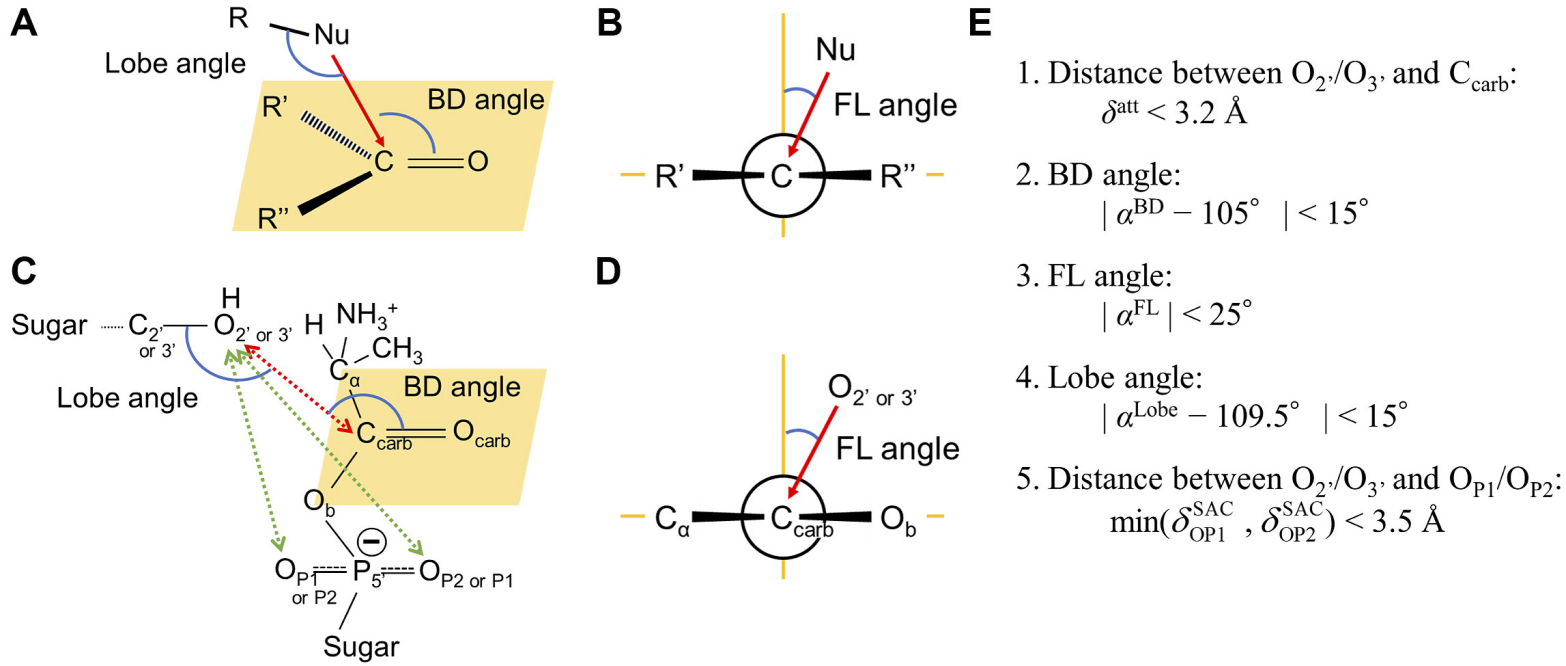
Angles and distances defining the geometry for the aminoacylation reaction. Definition of BD and Lobe angles are shown in (**A**), and FL angle is shown in (**B**) for the nucleophile (Nu) attacking the unsaturated carbonyl carbon atom. Five measures that define a reactive geometry in this study are shown in (**C**) and (**D**): BD, Lobe angles, distance between C_carb_---O_2′_/O_3′_ represented by the red dotted arrow, distances between O_2′_/O_3′_---O_P1_/O_P2_ represented by the green dotted arrows are shown in (C); FL angle is shown in (D). Five criteria used in this study are shown in (**E**).

In physical organic chemistry, the approaching geometry of a nucleophile (R-Nu-H) on a trigonal unsaturated *sp*^2^ carbon of carbonyl group (C=O) could be defined by one distance and three angles, as shown in Figure 3. Nu is the nucleophile, and R is the linked atom to the nucleophile. This system defines the distance between Nu and C, the Bürgi–Dunitz (BD) (30,31) and Flippin–Lodge (FL) (32) angles, and an angle related to the atomic orbital of Nu. The third angle has not been studied yet, and it is introduced here. For the sake of clarity, it is called the ‘lobe angle’. C and O correspond to an α-carboxylate carbon (C_carb_) and an α-carboxylate oxygen (O_carb_) of the aminoacyl phosphate, respectively.

#### Distance between Nu and C_carb_

The distance between Nu and C (*δ*^att^) is obviously the most important factor for the approach of Nu to the carbonyl carbon. We define the first requirement for the reactive geometry as follows:

(Criterion 1) \begin{equation*}{\delta ^{{\rm{att}}}} < 3.2\, \mathring{\rm A}.\end{equation*}

#### BD angle

The BD angle (*α*^BD^) is defined as the Nu···C=O angle (31). The BD angle has a preference of ∼105°, which was found by a survey of crystal structures of compounds containing nucleophilic groups and electrophilic centers (31). This angle preference was also shown in quantum mechanical calculations for simple model systems (30). Similar to other studies described above, *α*^BD^ is also expected to be ∼105°. We define the second requirement for the reactive geometry as follows:

(Criterion 2) \begin{equation*}|{\alpha ^{{\rm{BD}}}}-105^\circ | < 15^\circ .\end{equation*}

#### FL angle

The FL angle (*α*^FL^) is defined as the angle between the plane formed by Nu, C and O atoms and that perpendicular to the carbonyl plane formed by C, O and R’ (R’’), where R’ and R’’ are the carbonyl substituents (32). A nucleophile must approach a carbonyl carbon while staying further away from the two carbonyl substituents. Therefore, preference of the FL angle depends on the relative steric size of the two substituents attached to the carbonyl carbon. For a symmetric substitution case (R’ = R’’), the FL angle is simply expected to be ∼0°. In the case described here, R’ (R’’) corresponds to the *α*-carbon of alanine (C_α_) and the bridging phosphate oxygen (O_b_) when just focusing on the neighboring atom. However, the van der Waals radii of O (1.52 Å) and C (1.7 Å) are slightly different (33), and *α*^FL^ would be close to 0°. (The R’ substituent is sterically bulkier than just a C_α_ atom alone in this model, so the FL angle should be measured in a clockwise direction.) We defined the third requirement for the reactive geometry as follows:

(Criterion 3) \begin{equation*}\left| {{\alpha ^{{\rm{FL}}}}} \right| < 25^\circ .\end{equation*}

#### Lobe angle

The lobe angle (*α*^Lobe^) is defined here as the angle formed by R−Nu···C. The angle would also be an important factor for its reactivity since the HOMO of the nucleophile has a specific geometry. In aminoacylation of the RNA minihelix, hydroxyl oxygen as a nucleophile has the *sp*^3^ hybrid orbital so that *α*^Lobe^ should be also restricted to ∼109.5°. We defined the forth requirement for the reactive geometry as follows:

(Criterion 4) \begin{equation*}|{\alpha ^{{\rm{Lobe}}}}-109.5^\circ | < 15^\circ .\end{equation*}

#### Distance between Nu and non-bridging phosphate oxygens

We obtained an additional constraint from the SAC model that defines the possible reactive geometry. The 3′-OH group in the terminal adenosine is thought to be deprotonated for attacking the α-carboxylate carbon in aminoacylated thymine. Therefore, Nu should be close to one of the non-bridging phosphate oxygens (O_P1_ and O_P2_). We describe these distances of Nu-O_P1_ and Nu-O_P2_ as $\delta _{{\rm{OP1}}}^{{\rm{SAC}}}$ and $\delta _{{\rm{OP2}}}^{{\rm{SAC}}}$, respectively, and defined the fifth requirement for the reactive geometry as follows:

(Criterion 5) \begin{equation*}min({\delta _{{\rm{OP1}}}^{{\rm{SAC}}}, \delta _{{\rm{OP2}}}^{{\rm{SAC}}})} < 3.5\, \mathring{\rm A}.\end{equation*}

Here, the cutoff distance of 3.5 Å is the diameter of an oxygen atom (3.0 Å (33)) plus a buffer length (0.5 Å).

In this paper, we defined the conformation obtained from the MD simulation as ‘reactive’ if the conformation satisfied the five criteria described above regarding the geometry at the reaction site. Hereafter, the conformation with reactive geometry is called a ‘reactive conformation.’ As all MD simulations in this study were performed without any biased potential to force O_3′_ as Nu, the MD trajectories are evaluated with (Nu, R) = (O_2′_, C_2′_) as well as (Nu, R) = (O_3′_, C_3′_).

## RESULTS

### Number of reactive conformations

We evaluated how often the modeled RNA minihelix exhibited the reactive conformation in the MD simulations of the L-Ala and D-Ala systems using 120 000 structures for each system. These numbers of structures are listed in Table 1. In the case of (Nu, R) = (O_3′_, C_3′_), 145 and 48 structures adopted the reactive conformation in the L-Ala and D-Ala systems, respectively. The difference was statistically significant with *P*-value of 0.03 (Welch’s unpaired *t*-test). The 3:1 ratio agrees qualitatively with the 4:1 activity ratio determined by experiments (5). In the case of (Nu, R) = (O_2′_, C_2′_), 5 and 1 structures adopted the reactive conformation in the L-Ala and D-Ala systems, respectively. The number of reactive conformations with O_2′_ as Nu is so small as to be negligible compared with those with O_3′_ as Nu, which is also consistent with the experimental results. The numbers of reactive conformations meeting the criteria with various distances and angles are listed in Table S1. Even using different buffer values, we obtained the qualitatively same results. In the case of (Nu, R) = (O_3′_, C_3′_), the use of criteria without FL angle (bottom row in Table S1) selected the almost same numbers of reactive conformations in the L-Ala and D-Ala systems, 149 and 49 structures in the L-Ala and D-Ala systems, respectively, as the original criteria (top row in Table S1). Averaged FL angle over obtained 149 reactive conformations of L-Ala was 3.1 ± 11°. Therefore, constrains of O_3′_-C_carb_ distance, BD angle, lobe angles and O_3′_-O_P1_/O_P2_ distance necessarily lead FL angle of ∼0°. These results strongly indicate that the trajectories obtained by the classical MD simulations contain useful information revealing chiral-selective mechanisms of the RNA minihelix aminoacylation.

**Table 1.** Number of reactive conformations with (Nu, R) = (O_3′_, C_3′_) and (O_2′_, C_2′_) found in 120 000 structures sampled from trajectories of the L-Ala and D-Ala systems

System	(Nu, R) = (O_3′_, C_3′_)*	(Nu, R) = (O_2′_, C_2′_)
L-Ala	145	5
D-Ala	48	1

*p-value calculated using the Welch’s unpaired *t*-test is 0.03.

### Backbone structure of the reactive conformation

Table 2 lists the average number of Watson–Crick hydrogen bonds in base pairs near the active site (A_3_-U_10_ and dT_4_-A_9_) and the averaged root-mean-square deviation (RMSD) values for heavy atoms in the two base pairs from the ideal A-form RNA double helical structure for reactive conformations of the L-Ala and D-Ala systems. In the reactive conformations for both systems, A_3_-U_10_ and dT_4_-A_9_ formed nearly two canonical hydrogen bonds in each base pair. Averaged RMSD values for the reactive conformations were lower than 1.6 Å. These results indicate that the structures surrounding the active site in the reactive conformations are close to the ideal A-form RNA double helix.

**Table 2.** Average number of Watson–Crick hydrogen bonds in the A_3_-U_10_ and dT_4_-A_9_ base pairs and average RMSD values of heavy atoms in the two base pairs from the ideal A-form RNA/DNA double helical structure for reactive conformations in the L-Ala and D-Ala systems

System	(Nu, R)	Average number of Watson–Crick hydrogen bonds	Average RMSD (Å)
L-Ala	(O_3′_, C_3′_)	1.8 ± 0.4	1.1 ± 0.5
	(O_2′_, C_2′_)	1.8 ± 0.4	1.6 ± 0.5
D-Ala	(O_3′_, C_3′_)	1.9 ± 0.2	0.9 ± 0.2
	(O_2′_, C_2′_)	2.0*	1.3*

*The number of samples in this class is one.

In the A-form RNA, the distance between the O of the 3′-OH of the ribose of A_3_ and the C_carb_ covalently connected to 5′-phosphate is shorter than the distance between O of the 2′-OH and C_carb_. Thus, the probability of forming a reactive geometry with O_3′_ as Nu is much higher than that with O_2′_ in the model.

### The physico-chemical origin of chiral preference for L-Ala over D-Ala in the reaction

**Figure 4.**
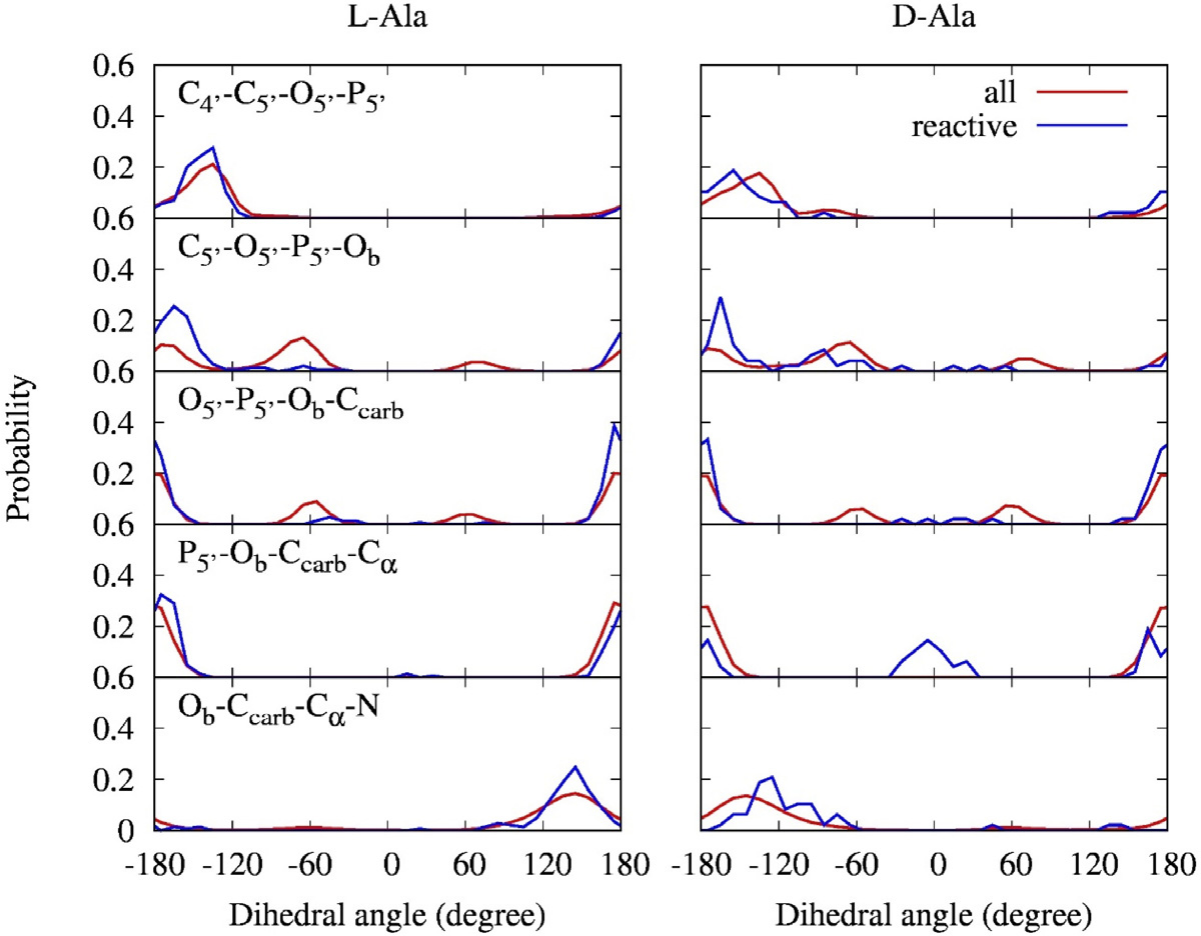
Probability distributions of dihedral angles in the alanyl phosphate calculated from all conformations (red lines) and reactive conformations (blue lines). Four atoms defining dihedral angles are shown only in the graphs of L-Ala on the left. The graphs on the same row use the same dihedral angles.

To reveal chiral selectivity mechanisms for the RNA minihelix, we investigated the structural features of the alanyl phosphate. Figure 4 shows the distributions of dihedral angles *τ*(*i-j-k-l*) with four linked atoms *i, j, k* and *l* around alanyl phosphate calculated from all conformations and reactive conformations.

For dihedral angles calculated from all conformations (Figure 4, red lines), the probability distributions of *τ*(C_4′_-C_5′_-O_5′_-P_5′_), *τ*(C_5′_-O_5′_-P_5′_-O_b_), *τ*(O_5′_-P_5′_-O_b_-C_carb_) and *τ*(P_5′_-O_b_-C_carb_-C_α_) for the L-Ala and D-Ala systems were nearly identical. On the other hand, the probability distributions of *τ*(O_b_-C_carb_-C_α_-N) for the L-Ala and D-Ala systems showed mirror images of one another reflecting the chirality of amino acid, where single peaks at +145° for L-Ala and −145° for D-Ala were observed.

Next, we analyzed the probability distributions of the dihedral angels of alanyl phosphate in the reactive conformations (Figure 4, blue lines). In the case of the L-Ala system, the probability distributions of all dihedral angles examined here showed strong individual peaks that sufficiently overlapped with those calculated from all conformations. This result indicates that the alanyl phosphate with reactive geometry for the L-Ala system likely has a preferential conformation consisting of a combination of the most probable dihedral angles. In the case of the D-Ala system, probability distributions of dihedral angles showed similar patterns as the L-Ala system except for *τ*(P_5′_-O_b_-C_carb_-C_α_), which exhibited two clear peaks at ∼180° and ∼0°. The former peak of *τ*(P_5′_-O_b_-C_carb_-C_α_) = ∼180° was the same as in the L-Ala system, whereas the latter peak of *τ*(P_5′_-O_b_-C_carb_-C_α_) = ∼0° was distinctive for the D-Ala system. However, the corresponding peak was not found in the probability distribution calculated from all conformations, indicating that the conformation with *τ*(P_5′_-O_b_-C_carb_-C_α_) = ∼0° is thermodynamically–unstable, in contrast to the case where *τ*(P_5′_-O_b_-C_carb_-C_α_) = ∼180°. Nearly all *τ*(O_b_-C_carb_-C_α_-N) distributions in the reactive conformations for the L-Ala and D-Ala systems were near +145° and −145°, respectively, except for a single outlier found in the D-Ala system: *τ*(O_b_-C_carb_-C_α_-N) of 47.9°.

**Figure 5.**
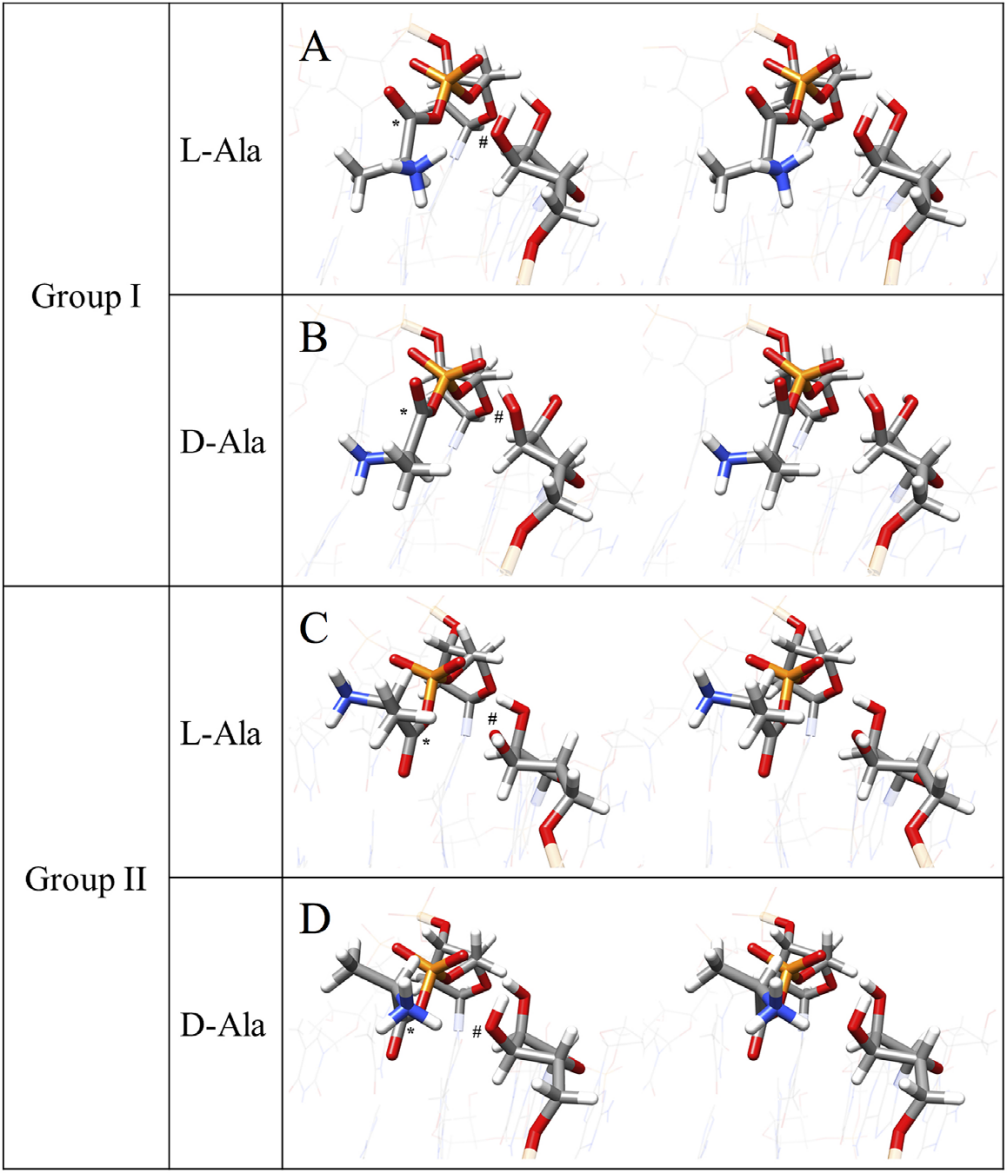
Representative structures of groups I and II for the L-Ala and D-Ala systems. The structures are shown as a cross-eyed stereo view. (**A**) Group I for L-Ala. (**B**) Group I for D-Ala. (**C**) Group II for L-Ala. (**D**) Group II for D-Ala. Ribose of A_3_, deoxyribose, phosphate and alanine moiety of dT_4_ are shown by a stick model and the others in nucleotides are shown by a wire frame model with transparent color. The elements are colored in the standard CPK rules: carbon, gray; hydrogen, white; oxygen, red; nitrogen, blue; phosphorus, orange. Carbonyl carbon (C_carb_) and 3′-oxygen (O_3′_) are marked by * and #, respectively.

To investigate the origin that causes the difference between the *τ*(P_5′_-O_b_-C_carb_-C_α_) distributions in the L-Ala and D-Ala systems, we checked the 3D structures of the reactive conformations. Reactive conformations satisfying |*τ*(C_5′_-O_5′_-P_5′_-O_b_)| ≥ 120°, |*τ*(O_5′_-P_5′_-O_b_-C_carb_)| ≥ 120° and |*τ*(P_5′_-O_b_-C_carb_-C_α_)| ≥ 120° for the L-Ala and D-Ala systems were grouped (groups I-L and I-D, respectively), resulting in 118 and 8 members, respectively (Table S2). The fractions of reactive conformations in groups I-L and I-D were 81 and 17%, respectively. The structures in this group I correspond to conformations with the most probable regions of dihedral angles (see Figure 4). Their representative structures with the lowest cumulative RMSD of all atoms in A_3_, alanine and dT_4_ with respect to every other structure in each group are shown in Figure 5A and B. All reactive conformations that belong to group I are shown in Supplementary Figure S1. The structures around the active sites in group I for the L-Ala and D-Ala systems were similar, where O_P1_, O_P2_ and O_carb_ atoms were placed on the same side with respect to the plane including both P_5′_ and C_carb_, and orthogonal to the carbonyl plane: *τ*(P_5′_-O_b_-C_carb_-O_carb_) = ∼0°. A distinct difference between the two structures is the position of the amino group of alanine relative to the O_3_′ nucleophile and the carbonyl group. For group I-L, the amino group and O_3_′ atom were placed on the same side with respect to the carbonyl plane, which was within the most probable dihedral angle for *τ*(O_b_-C_carb_-C_α_-N) for the L-Ala system, i.e. around +145°. For group I-D, the amino group was placed on the opposite side with respect to the carbonyl plane and methyl group of alanine was placed close to the O_3′_ atom. The most probable dihedral angle for *τ*(O_b_-C_carb_-C_α_-N) for the D-Ala system was around −145°. Van der Waals radii of the amino and methyl groups are estimated to be 1.64 and 1.88 Å, respectively (34). Therefore, the nucleophile O_3′_ could frequently approach the C_carb_ in the conformation of group I-L compared to group I-D conformation due to the difference of steric repulsion. Additionally, a hydrophilic hydroxyl group approaching to a hydrophilic amino group would require less energy than an approach to a hydrophobic methyl group. Electrostatic interaction between the relatively high electronegative hydroxyl group and positively charged amino group would also assist the approaching shown in group I-L. The observed difference in the number of members between groups I-L and I-D (118 versus 8) would reflect the size and hydrophilicity/electrostatic differences between groups close to the O_3′_ atom in the active site.

To examine the reactive conformations with *τ*(P_5′_-O_b_-C_carb_-C_α_) = ∼0°, which was characteristic of the D-Ala system with unstable angle (see Figure 4), reactive conformations satisfying |*τ*(C_5′_-O_5′_-P_5′_-O_b_)| ≥ 120° and |*τ*(O_5′_-P_5′_-O_b_-C_carb_)| ≥ 120°, and |*τ*(P_5′_-O_b_-C_carb_-C_α_)| ≤ 60° were grouped as group II. The number of structures in group II was 3 for the L-Ala system (group II-L) and 18 for the D-Ala system (group II-D), which correspond to fractions of 2 and 38% in the obtained reactive conformations, respectively (Table S2). Their representative structures are shown in Figure 5C and D. All reactive conformations that belong to group II are shown in Supplementary Figure S1. For group II, O_P1_ and O_P2_ atoms were placed on the same side with respect to the plane including both P_5′_ and C_carb_, and orthogonal to the carbonyl plane, but the O_carb_ atom was placed on the opposite side: *τ*(P_5′_-O_b_-C_carb_-O_carb_) = ∼180°. The methyl group of alanine was located close to O_3′_ in the L-Ala system (group II-L shown in Figure 5C), while the amino group of alanine was located close to O_3′_ in the D-Ala system (group II-D shown in Figure 5D). Thus, for a given conformation with |*τ*(C_5′_-O_5′_-P_5′_-O_b_)| and |*τ*(O_5′_-P_5′_-O_b_-C_carb_)| of ∼180° and *τ*(P_5′_-O_b_-C_carb_-C_α_) of ∼0°, the frequency required to adopt the reactive conformation in the D-Ala system would be higher than that in the L-Ala system. This is consistent with the observed number of conformations in groups II-L and II-D (3 versus 18).

**Figure 6.**
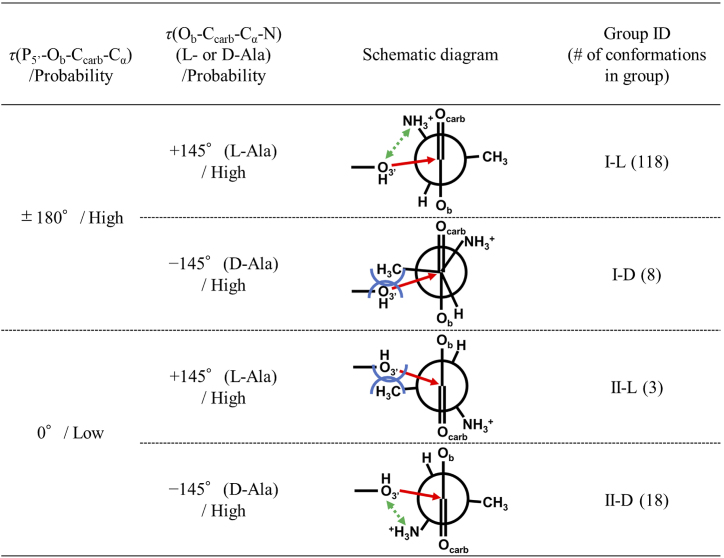
Proposed chiral-selective mechanism of aminoacylation of the RNA minihelix. In schematic diagram, steric repulsion between O_3′_ and CH_3_ is represented by blue arcs and electrostatic interaction between O_3′_ and NH_3_^+^ is represented by green broken arrow.

Figure 6 shows a summary of the chiral-selective mechanism of aminoacylation of the RNA minihelix. The number of reactive conformations was classified into eight groups according to the dihedral angles around alanyl phosphate. *τ*(C_5′_-O_5′_-P_5′_-O_b_), *τ*(O_5′_-P_5′_-O_b_-C_carb_) and *τ*(P_5′_-O_b_-C_carb_-C_α_) are listed in Table S2. All representative reactive conformations for eight groups are also shown in Figures S1 and S2, respectively.

## DISCUSSION

L-amino acid specific tRNA aminoacylation by aaRSs is a crucial step in modern translation. Once L-amino acid charged tRNAs are formed, they are used to synthesize proteins composed of L-amino acids (8). Modern aaRSs are highly specific in the incorporation of cognate (L-)amino acid because they have specific active sites for substrates, i.e. cognate (L-)amino acid, cognate tRNA and adenosine triphosphate. In addition, most of the aaRSs possess an editing site as well as an acylation site to hydrolyze misacylated tRNA (25). By engineering the active site of aaRSs, a wide range of non-canonical amino acids have also been incorporated into proteins in living cells (35). Computational study showed that the discrimination of L- and D-amino acids is controlled by a network of intermolecular interactions between aaRS and substrates (36).

Tamura and Schimmel handled the discrimination of L- and D-amino acids in a simplified situation without such elaborated active sites of aaRSs (Figure 1) (4). In aminoacylation of RNA minihelix, trigonal–tetrahedral interconversion at a carbonyl center may be important in chiral selectivity. However, Tamura and Schimmel showed that no aminoacylation was detected when the minihelix pretreated with NaIO_4_ was oxidized the *cis*-hydroxyl group of the 3′-terminal adenosine residue (4). Furthermore, no significant difference in reactivity was observed when the hydrolysis of L-Ala- and D-Ala-phosphate oligonucleotide was compared either in the presence or absence of the bridging oligonucleotide using the periodate oxidized minihelix (4). Therefore, the probability of properly approaching nucleophile O_3′_ to the trigonal carbonyl carbon of the aminoacyl group would be the most crucial in determining the chiral selectivity of this system.

To evaluate the possibility that the chemical reaction occurs in the framework of classical MD simulations, we have defined five geometric criteria for active sites such that the modeled RNA minihelix conformation should satisfy the reaction requirements based on physical organic chemistry principles and the SAC mechanism. Examples of SAC include not only aaRSs but also many other enzymes, such as serine protease, type II restriction endonuclease, GTPases and lysozymes (29). In the case of aaRSs, non-bridging aminoacyl-adenylate phosphate oxygens can act as the base to deprotonate the hydroxyl groups of the terminal adenosine of tRNA, 2′-OH for class I and 3′-OH for class II. The deprotonation mechanisms have been extensively examined by experiment for class I GlnRS by Steitz and co-workers (37,38), and for class II AspRS by Moras and co-workers (39,40), and for class II HisRS by Francklyn and co-workers (41). Especially, Francklyn and co-workers showed that substitution of the non-bridging Sp oxygen of the adenylate by sulfur decreases the transfer rate by at least 10 000-fold, providing strong experimental evidence for oxygen functioning as a general base in the reaction. A quantum mechanics calculation in HisRS showed that when the non-bridging Sp oxygen acts as a general base, the reaction yields the lowest-energy pathway compared with those in reactions where the bridging or non-bridging Rp phosphate oxygens act as a base (26).

If we assumed that O_3′_ acts as a nucleophile, the number of conformations with reactive geometry in the L-Ala system was much higher than that in the D-Ala system. In a reactive geometry, nucleotide structures near the active site were close to that of the ideal A-form RNA double helix, and the dihedral angles of backbone of alanine phosphate ester were within the most probable regions. In the canonical form, the distance between C_carb_ and O_3′_ was naturally much shorter than that between C_carb_ and O_2′_. In addition, when the O_3′_ atom approached the C_carb_ atom, the amino group of L-Ala and O_3′_ atom were placed on the same side with respect to the carbonyl plane. On the other hand, the methyl group in D-Ala and O_3′_ atom were placed on the same side with respect to carbonyl plane. Due to the smaller volume and hydrophilic/electrostatic nature of amino group, O_3′_ atom in the L-Ala system would approach the C_carb_ atom much more frequently than in the D-Ala system. In experiments, the chiral preference for L-amino acid in the RNA minihelix was observed not only for Ala but also Leu and Phe with ∼4-fold selectivity (4). Substitution of amino acid in the RNA minihelix would primarily affect the probability distribution of *τ*(O_b_-C_carb_-C_α_-N), which was an important factor to determine the chiral-selectivity shown above. This angle corresponds to the dihedral angle *ψ* for (N-C_α_-C-N) polypeptides. In the L-Ala system, *τ*(O_b_-C_carb_-C_α_-N) was dominantly clustered ∼145°, which explains why the methyl group remains distant from O_3′_ approaching C_carb_ in order to minimize steric clashes between them in the group I-L conformation. Spectroscopic analysis using nuclear magnetic resonance with polarized Raman, Fourier transform infrared and vibrational circular dichroism spectroscopies shows that the *ψ* angles of amino acids in Gly-X-Gly tripeptides (X = Ala, Val, Phe, Ser, Glu, Leu and Met) are substantially (84% on average) populated around the center with ∼150° (42). Therefore, the chiral-selective mechanism found in the present study using the Ala system would naturally explain that Leu and Phe have the same chiral-preference as Ala.

In summary, chiral selectivity for the aminoacylation reaction found in the RNA minihelix could be accomplished by simple principles: preferences of dihedral angles of the aminoacyl phosphate, chemical natures of the amino group and amino acid side chain. In future work, the details of the geometric and energetic changes along the reaction coordinates will be analyzed using the structures of RNA minihelix obtained in this study.

## Supplementary Material

Supplementary Data
