# Supplementary material for: Principles of chemical geometry underlying chiral selectivity in RNA minihelix aminoacylation
Source: Nucleic Acids Res. 2018 Oct 15;46(21):11144–52. doi: 10.1093/nar/gky909 (PMC6265448; doi:10.1093/nar/gky909)
Supplement: Supplementary Data [file gky909_supplemental_files.pdf]

Supplementary material to:

Principles of chemical geometry underlying chiral selectivity in RNA minihelix aminoacylation

Tadashi Ando[1,2,*], Shunichi Takahashi[1] and Koji Tamura[2,3,*]

[1]Department of Applied Electronics, Tokyo University of Science,
6-3-1 Niijuku, Katsushika-ku, Tokyo 125-8585, Japan
[2]Research Institute for Science and Technology, Tokyo University of Science,
2641 Yamazaki, Noda, Chiba 278-8510, Japan
[3]Department of Biological Science and Technology, Tokyo University of Science,
6-3-1 Niijuku, Katsushika-ku, Tokyo 125-8585, Japan

[*]To whom correspondence may be addressed. Email: tando@rs.tus.ac.jp or koji@rs.tus.ac.jp.

Contents

Table S1
Table S2
Figure S1
Figure S2

Table S1

Table S1. Number of reactive conformations found in the trajectories of the L-Ala and D-Ala systems using various distances and angles for geometrical criteria with (Nu, R) = ($O_{3'}$, $C_{3'}$)

Conditions	Number of conformations		Ratio (L-Ala/D-Ala)
	L-Ala	D-Ala	
$\delta^{att} < 3.2$ Å, $\lvert\alpha^{BD} - 105°\rvert < 15°$, $\lvert\alpha^{FL}\rvert < 25°$, $\lvert\alpha^{Lobe} - 109.5°\rvert < 15°$, $\min(\delta^{SAC}_{OP1}, \delta^{SAC}_{OP2}) < 3.5$ Å	145	48	3.0
$\delta^{att} < 3.2$ Å, $\lvert\alpha^{BD} - 105°\rvert < 15°$, $\lvert\alpha^{FL}\rvert < 15°$, $\lvert\alpha^{Lobe} - 109.5°\rvert < 15°$, $\min(\delta^{SAC}_{OP1}, \delta^{SAC}_{OP2}) < 3.5$ Å	121	35	3.5
$\delta^{att} < 3.0$ Å, $\lvert\alpha^{BD} - 105°\rvert < 15°$, $\lvert\alpha^{FL}\rvert < 25°$, $\lvert\alpha^{Lobe} - 109.5°\rvert < 15°$, $\min(\delta^{SAC}_{OP1}, \delta^{SAC}_{OP2}) < 3.5$ Å	47	9	5.2
$\delta^{att} < 3.5$ Å, $\lvert\alpha^{BD} - 105°\rvert < 15°$, $\lvert\alpha^{FL}\rvert < 25°$, $\lvert\alpha^{Lobe} - 109.5°\rvert < 15°$, $\min(\delta^{SAC}_{OP1}, \delta^{SAC}_{OP2}) < 3.5$ Å	235	88	2.7
$\delta^{att} < 3.2$ Å, $\lvert\alpha^{BD} - 105°\rvert < 25°$, $\lvert\alpha^{FL}\rvert < 25°$, $\lvert\alpha^{Lobe} - 109.5°\rvert < 15°$, $\min(\delta^{SAC}_{OP1}, \delta^{SAC}_{OP2}) < 3.5$ Å	177	84	2.1
$\delta^{att} < 3.2$ Å, $\lvert\alpha^{BD} - 105°\rvert < 15°$, $\lvert\alpha^{FL}\rvert < 25°$, $\lvert\alpha^{Lobe} - 109.5°\rvert < 25°$, $\min(\delta^{SAC}_{OP1}, \delta^{SAC}_{OP2}) < 3.5$ Å	245	169	1.4
$\delta^{att} < 3.2$ Å, $\lvert\alpha^{BD} - 105°\rvert < 25°$, $\lvert\alpha^{FL}\rvert < 25°$, $\lvert\alpha^{Lobe} - 109.5°\rvert < 25°$, $\min(\delta^{SAC}_{OP1}, \delta^{SAC}_{OP2}) < 3.5$ Å	327	306	1.1
$\delta^{att} < 3.2$ Å, $\lvert\alpha^{BD} - 105°\rvert < 15°$, $\lvert\alpha^{FL}\rvert < 25°$, $\lvert\alpha^{Lobe} - 109.5°\rvert < 15°$, $\min(\delta^{SAC}_{OP1}, \delta^{SAC}_{OP2}) < 4.0$ Å	309	135	2.3
$\delta^{att} < 3.2$ Å, $\lvert\alpha^{BD} - 105°\rvert < 180°$, $\lvert\alpha^{FL}\rvert < 180°$, $\lvert\alpha^{Lobe} - 109.5°\rvert < 180°$, $\min(\delta^{SAC}_{OP1}, \delta^{SAC}_{OP2}) < 99$ Å	3653	4289	0.9
$\delta^{att} < 3.2$ Å, $\lvert\alpha^{BD} - 105°\rvert < 15°$, $\lvert\alpha^{FL}\rvert < 180°$, $\lvert\alpha^{Lobe} - 109.5°\rvert < 180°$, $\min(\delta^{SAC}_{OP1}, \delta^{SAC}_{OP2}) < 99$ Å	2144	2157	1.0
$\delta^{att} < 3.2$ Å, $\lvert\alpha^{BD} - 105°\rvert < 15°$, $\lvert\alpha^{FL}\rvert < 25°$, $\lvert\alpha^{Lobe} - 109.5°\rvert < 180°$, $\min(\delta^{SAC}_{OP1}, \delta^{SAC}_{OP2}) < 99$ Å	2023	2009	1.0
$\delta^{att} < 3.2$ Å, $\lvert\alpha^{BD} - 105°\rvert < 15°$, $\lvert\alpha^{FL}\rvert < 180°$, $\lvert\alpha^{Lobe} - 109.5°\rvert < 15°$, $\min(\delta^{SAC}_{OP1}, \delta^{SAC}_{OP2}) < 99$ Å	723	564	1.3
$\delta^{att} < 3.2$ Å, $\lvert\alpha^{BD} - 105°\rvert < 15°$, $\lvert\alpha^{FL}\rvert < 180°$, $\lvert\alpha^{Lobe} - 109.5°\rvert < 15°$, $\min(\delta^{SAC}_{OP1}, \delta^{SAC}_{OP2}) < 3.5$ Å	149	49	3.0

Table S2

Table S2. Number of reactive conformations in eight groups classified by dihedral angles of alanyl phosphate

Dihedral angles (°)			Number of conformations*		Group ID
τ($C_{5'}$-$O_{5'}$-$P_{5'}$-O_b)	τ($O_{5'}$-$P_{5'}$-O_b-C_{carb})	τ($P_{5'}$-O_b-C_{carb}-C_α)	L-Ala	D-Ala	
≥ 120	≥ 120	≥ 120	118 (0.81)	8 (0.17)	I
		≤ 60	3 (0.02)	18 (0.38)	II
	< 120	≥ 120	12 (0.08)	4 (0.08)	III
		≤ 60	0 (0)	0 (0)	
< 120	≥ 120	≥ 120	12 (0.08)	10 (0.21)	IV
		≤ 60	0 (0)	7 (0.15)	V
	< 120	≥ 120	0 (0)	1 (0.02)	VI
		≤ 60	0 (0)	0 (0)	

*Fractions of groups are shown in parenthesis.

Figure S1

Group ID	L-Ala	D-Ala
I	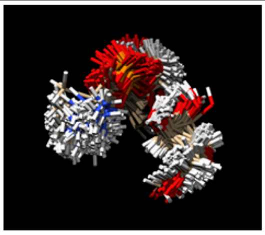	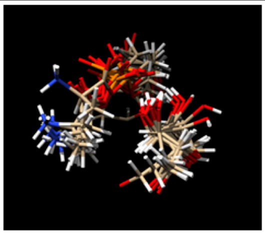
II	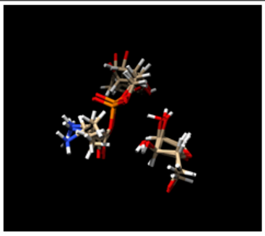	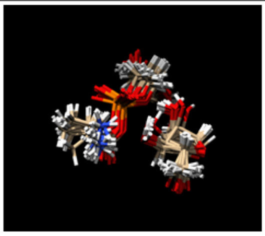
III	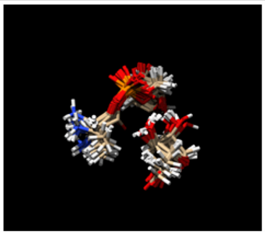	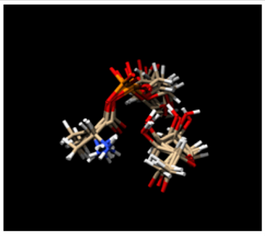
IV	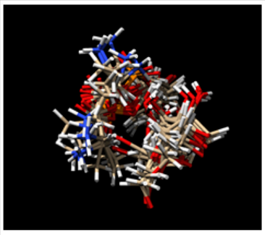	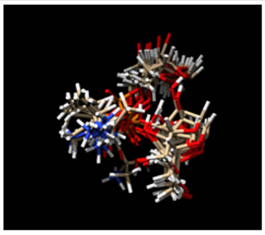
V	-	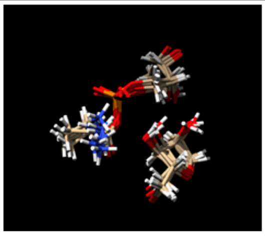
VI	-	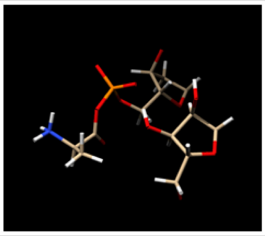

Figure S1. Structures of members in eight groups listed in Table S2. Structures in group IV for L-Ala were not well clustered due to heterogeneity in $\tau(C_{4'}\text{-}C_{5'}\text{-}O_{5'}\text{-}P_{5'})$ and $\tau(O_b\text{-}C_{carb}\text{-}C_\alpha\text{-}N)$.

Figure S2

Group ID	L-Ala	D-Ala
I	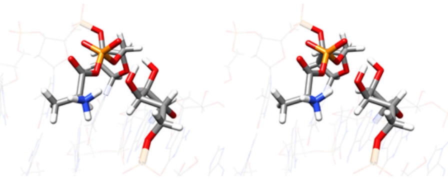	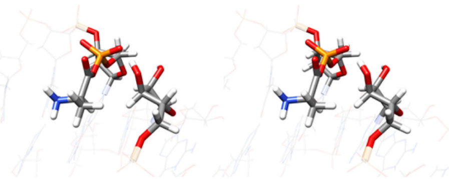
II	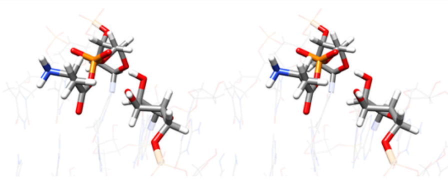	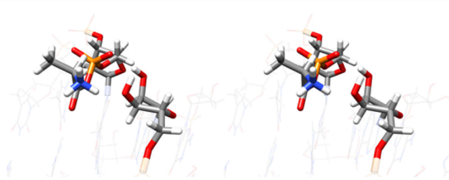
III	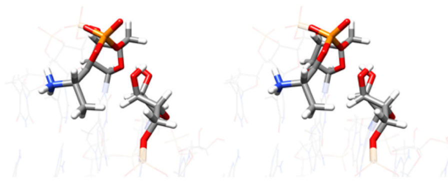	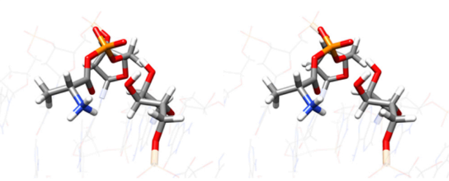
IV	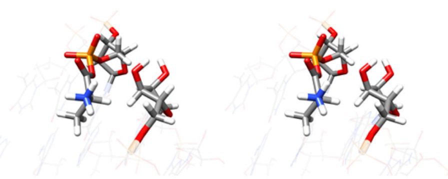	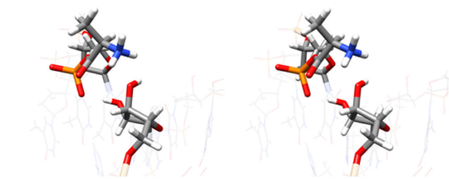
V	-	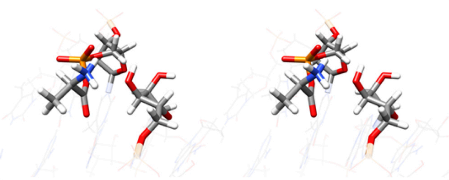
VI	-	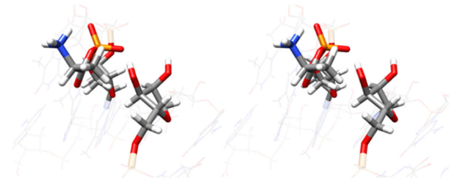

Figure S2. Representative structures of eight groups listed in Table S2. The structures are shown as a cross-eyed stereo view.
